# 

**Published:** 1964-01

**Authors:** 


					f I ^ -?" "T January 1904
MedicoChiru
Journ
X?* r_^..
index medscus
JTC  . . A 5? -
:|vOL-!SSUE
? INDEXER. ?i5...
?RPORATING the medical journal of the south west
Vol 79 (i) NO 291

				

